# Supplementary material for: 3D virtual reality vs. 2D desktop registration user interface comparison
Source: PLoS One. 2021 Oct 27;16(10):e0258103. doi: 10.1371/journal.pone.0258103 (PMC8550408; doi:10.1371/journal.pone.0258103)
Supplement: S1 Appendix — (DOCX) [file pone.0258103.s001.docx]

PLOS ONE Supporting Information Appendix S1

Article title: 3D Virtual Reality vs. 2D Desktop Registration User Interface Comparison

Andreas Bueckle^1*^, Kilian Buehling^2^, Patrick C. Shih^3^, Katy Börner^1,4^

^1^ Department of Intelligent Systems Engineering, Luddy School of Informatics, Computing, and Engineering, Indiana University, Bloomington, Indiana, United States of America

^2^ Research Group Knowledge and Technology Transfer, Fakultät Wirtschaftswissenschaften, Technische Universität Dresden, Germany

^3^ Department of Informatics, Luddy School of Informatics, Computing, and Engineering, Indiana University, Bloomington, Indiana , United States of America

^4^ Department of Information and Library Science, Luddy School of Informatics, Computing, and Engineering, Indiana University, Bloomington, Indiana, United States of America

* Corresponding author

Email: [abueckle@iu.edu](mailto:abueckle@iu.edu) (AB)

The following Supporting Information is available for this article:

- Supplementary text
- Tables S1 to S2

Other supplementary materials for this manuscript include:

- Qualtrics survey PDF: <https://github.com/cns-iu/rui-tissue-registration/blob/main/RUI_user_study_questionnaires.pdf>
- Video demonstrations of three setups (2D Desktop, VR Tabletop, VR Standup):
  - [2D Desktop](https://iu.mediaspace.kaltura.com/media/t/1_kz5c7l9m)
  - [VR Tabletop](https://iu.mediaspace.kaltura.com/media/t/1_yd8dxogy)
  - [VR Standup](https://iu.mediaspace.kaltura.com/media/t/1_bnr5wpcd)

These materials are hosted on GitHub: <https://github.com/cns-iu/rui-tissue-registration>

# Power Analysis

We performed a power analysis to estimate a sample size that would give us a high chance of statistically significant results. The sample size calculation was performed using G*Power [1], an open-source statistical power analysis tool developed at the University of Duesseldorf in Germany. Among other functionality, G*Power allows the user to set a range of metrics to retrieve a sample size for a given research design as expressed through the metrics outlined below. Before being able to use G*Power, we needed to parametrize our study.

The user study presented in our paper was the first in a series of similar studies. Thus, an essential factor when parametrizing this research design for sample size calculation was that it contained two separate user studies that differed by the number of groups (user study #1 (in this paper): 3 setups = 3 groups; user study #2 (not described here): 3 setups x 2 cohorts = 6 groups), number of measurements, and thus the required sample size (see **Table S1**). For the subsequent user study (discussed elsewhere, in progress), we used the data from our RUI study described here as control cohort.

The statistical power calculation also needed other parameters from the researcher:

- **Test family**
  - We chose “F tests” since it allowed us to perform ANOVA for a comparison of repeated measures between groups of subjects
- **Statistical test**
  - The specific test we chose to perform was a between-factors, repeated-measures ANOVA since we aimed to analyze between subjects/users (as opposed to just within subjects—i.e., just within users)
- **Type of power analysis**
  - G*Power allowed us to compute a variety of power analysis metrics. We chose “A priori: Compute required sample size” in order to determine the number of subjects needed for this study
- **Effect size**
  - The effect size is defined as the quotient of the mean difference over the standard deviation. In G*Power, the effect size can range from 0 to 50. It is always positive or zero. The default effect size in G*Power was set to 0.25. We chose 0.3 for our effect size calculation.
  - Literature [2, 3] suggests that it is common to distinguish between small (0.2), medium (0.5) and high (0.8) effect sizes in power analysis, so choosing 0.3 seemed reasonably conservative (as sample size is negatively correlated with effect size, *ceteris paribus*). Effect size is commonly seen as either small, medium, or large. Ideally, the effect size can be estimated by looking at pilot study data. The pilot study data at hand, however, may not be sufficient in order to produce an accurate result for this estimate.
- **Alpha error probability**
  - Probability of Type-I error
  - Our assumed alpha error probability of 0.05, which is a standard value.
- **Power**
  - 1 - Probability of Type-II error (beta)
  - Standard value for beta is usually set to four times the alpha error probability
  - We set the power metric to 0.8.

Given these values for the metrics required by G*Power, we obtained the results shown in **Table S1**. As mentioned above, please note that we conducted a separate user study described in a forthcoming paper where we compared two cohorts of 2D Desktop, VR Tabletop, and VR Standup users (a control and an experiment cohort). This is why we chose 6 as the “number of groups” for the Plateau phase as we were interested in comparing performances between the control and experiment cohort. We took the resulting 84 subjects and divided them by 2. The users in this study (84/2 = n = 42) then served as control cohort for the forthcoming study.

**Table S1. Metrics for sample size calculation by phase.** Notice that all the values are identical except the number of groups and measurements.

| **Metrics** | **Ramp-Up** | **Plateau** |
| --- | --- | --- |
| **Effect size** | 0.3 | 0.3 |
| **Alpha error probability** | 0.05 | 0.05 |
| **Power** | 0.8 | 0.8 |
| **Number of groups** | 3 | 6 |
| **Number of measurements** | 14 | 30 |
| **Sample size** | 63 | 84 |

**Table S1** shows that the two studies contained in our RUI VR research design required two different sample sizes to achieve the targeted effect of size 0.3. Since we were conducting both studies together, we needed to aim for the higher number: **84 subjects, or 14 subjects per group per setup** (Control-VR Standup, Control-VR Tabletop, Control-2D Desktop, Experiment-VR Standup, Experiment-VR Tabletop, Experiment-2D Desktop).

# Data Collected from Unity

**Table S2. Telemetry data logged during experiment.**

| Name of metric | **Definition** | **Setup** |
| --- | --- | --- |
| elapsedTime | Number of seconds since the beginning of the experiment | All |
| headsetX, headsetY, headsetZ | Position of HMD, from the origin of the virtual scene (VR only), in meters | VR Tabletop, VR Standup |
| controllerLeftX, controllerLeftY, controllerLeftZ, controllerRightX, controllerRightY, controllerRightZ, | Positions of controllers, from the origin in the virtual scene (VR only), in meters | VR Tabletop, VR Standup |
| mousepositionX, mousepositionY | The user’s 2D mouse movements, in pixels | 2D Desktop |
| distance | Distance between the centroids of the two blocks, in meters (VR)/ Unity scene units (2D Desktop) | All |
| currentTissueBlock.transform.position.x, currentTissue block.transform.position.y, currentTissue block.transform.position.z | Current position of the tissue block | All |
| objectXLength, objectYLength, objectZLength | Current dimensions of tissue block (and target) | All |
| currentObjectRotationX, currentObjectRotationY,  currentObjectRotationZ | Current x, y, z rotation of tissue block | All |
| targetRotationX, targetRotationY,  targetRotationZ | Current x, y, z rotation of target block | All |
| angle | Current rotational difference between tissue block and target block, in degrees (0-180) | All |
| button | Current button pressed (“grab” and “menu” in VR, “left” or “right” mouse button on 2D Desktop), or function called (“reset position”, “reset rotation”, etc.) | All |
| side | “Left” or “right” hand (VR), “left” or “right” mouse button (2D Desktop) | VR |
| status | “Down” (pressed), “up” (released), “dragging” (2D Desktop only) | All |

# References

1. Faul F, Erdfelder E, Lang A-G, Buchner A. G* Power 3: A flexible statistical power analysis program for the social, behavioral, and biomedical sciences. Behavior Research Methods. 2007;39(2):175-191. doi: 10.3758/bf03193146

2. Brysbaert M. How many participants do we have to include in properly powered experiments? A tutorial of power analysis with reference tables. J Cogn. 2019;2(1):1-38. doi: 10.5334/joc.72

3. Cohen J. Statistical power analysis for the behavioral sciences. New York City, NY, USA: Academic Press; 2013.
